# Microbiome dataset of spontaneously fermented Ethiopian honey wine, *Tej*

**DOI:** 10.1016/j.dib.2022.108022

**Published:** 2022-03-14

**Authors:** Eskindir Getachew Fentie, Minsoo Jeong, Shimelis Admassu Emire, Hundessa Dessalegn Demsash, Min A Kim, Hwang-Ju Jeon, Sung-Eun Lee, Setu Bazie Tagele, Yeong-Jun Park, Jae-Ho Shin

**Affiliations:** a College of Biological and Chemical Engineering, Addis Ababa Science and Technology University, Addis Ababa 16417, Ethiopia; b Department of Applied Biosciences, Kyungpook National University, Daegu 41566, Korea; c School of Chemical and Bio-Engineering, Addis Ababa Institute of Technology, Addis Ababa University, P.O. Box 385, King George VI Street, Addis Ababa 16417, Ethiopia; d National Institute for Korean Medicine Development, Daegu, 38540, Korea

**Keywords:** Alpha diversity, Beta diversity, *Tej*, Linear discriminated analysis

## Abstract

This dataset contains raw and analyzed microbial data for the samples of spontaneously fermented Ethiopian honey wine, *Tej*, collected from three locations of Ethiopia. It was generated using culture independent amplicon sequencing technique. To gain a better understanding of microbial community variance and similarity across *Tej* samples from the same and different locations, the raw sequenced data obtained from the Illumina Miseq sequencer was subjected to a bioinformatics analysis. Lower diversity and richness of both bacterial and fungal communities were observed for all of the *Tej* samples. Besides, samples collected from Debre Markos area showed a significant discriminating tax for both bacterial and fungal communities. In nutshell, this amplicon sequencing dataset provides a useful collection of data for modernizing this spontaneous fermentation into a directed inoculated fermentation. Detail discussion on microbiome of *Tej* samples is given in [1].

## Specifications Table

**Table:** 

Subject	Biological Science
Specific subject area	Microbiome, spontaneously fermented beverage
Type of data	Table, Figure, FASTA file
How the data were acquired	Illumina MiSeq (Illumina-MiSeq-USA) platform were used for 16SrRNA and ITS amplicon sequencing. Besides, bioinformatic and statistical analysis were performed via QIIME2 and RStudio 4.0.3, respectively.
Data format	Raw, filtered and analysed
Description of data collection	The microbial DNA of all *Tej* samples were extracted, amplified, sequenced and analysed sequentially.
Data source location	A total 21 *Tej* samples were collected from Addis Ababa (lat. 8.9806, long. 38.7578), Bahir Dar (lat. 11.5742, long. 37.3614), Debre Markos (lat. 10.3296, long. 37.7344) areas The collected samples were analysed in: Kyungpook National University, Daegu, Korea,
Data accessibility	Repository name: National Center for Biotechnology Information (NCBI) Sequence Read Archive (SRA) data: Accession number PRJNA781236 and PRJNA781563 Direct URL to data: https://www.ncbi.nlm.nih.gov/Traces/study/?acc=PRJNA781236 and https://www.ncbi.nlm.nih.gov/Traces/study/?acc=PRJNA781563 Repository name: Science Data Bank Data identification number: 31253.11.sciencedb.01345 Direct URL to data: https://www.scidb.cn/en/s/URFf2q
Related research article	E. Fentie, M. Jeong, S. Emire, H. Demsash, M.A. Kim, H.J. Jeon, S.E. Lee, S. Tagele, Y.J. Park, J.H. Shin, Physicochemical properties, antioxidant activities and microbial communities of Ethiopian honey wine, Tej, Food Res. 152 (2022) 110765. https://doi.org/10.1016/j.foodres.2021.110765

## Value of the Data

• Helps to identify the dominant bacterial and fungal genus found in *Tej* samples.
• Helps to understand the differences and similarities of the microbial community structure for spontaneously fermented *Tej* samples.
• Helps on the development of direct *Tej* fermentation system.

## 1 Data

**Fig. 1:**
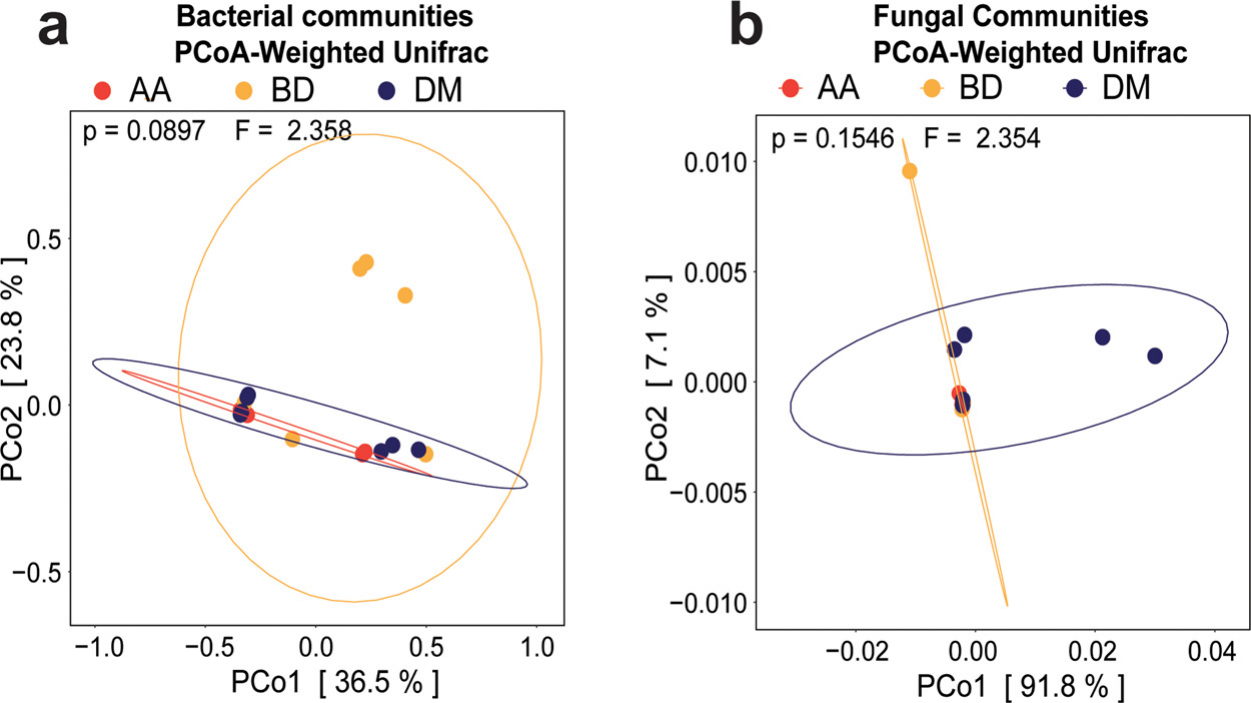
Principal co-ordinate analysis of weighted UniFrac distance (PCoA) plots demonstrating the beta diversity of **a**) bacterial and **b**) fungal communities. The dots on the plots represent the individual samples from respective areas. Red–Addis Ababa (AA), Orange–Bahir Dar (BD), Deep blue–Debre Markos (DM) samples.

**Fig. 2:**
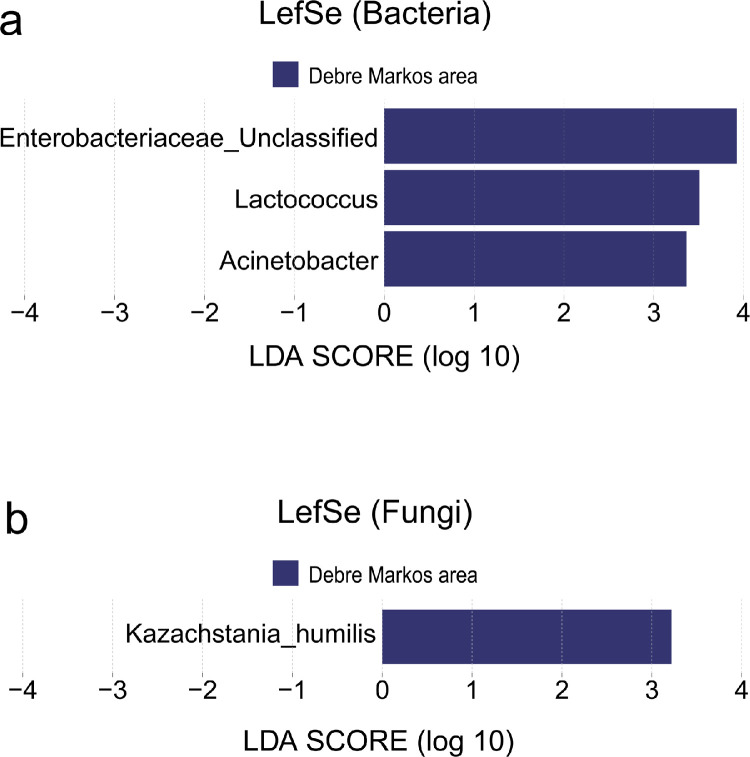
Linear discriminant analysis effect size (LefSe) for **a**) bacteria and **b**) fungi communities.

This dataset contains the microbiome data of both bacteria and fungi communities for *Tej* samples collected from three different locations of Ethiopia. The raw bacterial and fungal FASTA files of each sample are made accessible via National Center for Biotechnology Information (NCBI) data repository system. These FASTA files were the original metadata that were used for the bioinformatics analysis of this study. Table 1, describes the alpha diversity indices (Chao 1, Shannon, Simpson, Evenness, InvSimpson and observed) of each sample. This table is aimed to show the differences in alpha diversity indices based on sample collecting areas. Besides, Table 2 shows the list of bacterial and fungal communities that has less than 1% relative abundance. It showed all level of taxonomical classifications (Phylum, Class, Order, Family, and Genus) alongside its relative abundance of both bacterial and fungal communities. Both tables are made accessible on *Science Data Bank* data repository system. Furthermore, the quantitative bacterial and fungal beta diversity of the collected *Tej* samples was illustrated by using weighted-Unifrac principal coordinate analysis (PCoA) plot (Fig. 1). The relative abundance of each taxon for both bacterial and fungi communities from respective sample collection areas were the major comparing factor for microbial ecology diversity analysis. The distance metrics in the weighted-Unifrac PCoA plot demonstrated differences in microbial taxon abundance between the collected Tej samples (Fig. 1). Moreover, Fig. 2 demonstrate linear discriminant analysis effect size (LefSe) of bacteria and fungi for collected *Tej* samples based on the sample collection area. This figure was basically used to describe the significantly higher abundant bacterial and fungi taxon found in the grouped samples. Besides, all of the identified taxon in Fig. 2 were screened out using a linear discriminant analysis score of greater than 3.0. (Fig. 2).

**Table 1:** Alpha diversity of bacteria and fungi communities.

	Alpha diversity indices for bacteria						Alpha diversity indices for fungi					
Locations	Chao1	Shannon	Simpson	Evenness	Invsimp	Obs	Chao1	Shannon	Simpson	Evenness	Invsimp	Obs
A1	20	2.549232	0.902917	0.850955	10.30043	20	1	0	0	0	1.00	1
A2	11	1.817178	0.78934	0.757822	4.746981	11	2	0.000968	0.000189	0.001397	1.000189	2
A3	14	1.745972	0.772441	0.661589	4.39446	14	1	0	0	0	1.00	1
A4	7	0.623537	0.264767	0.320435	1.360114	7	1	0	0	0	1.00	1
A5	23	2.425679	0.882881	0.773619	8.53834	23	2	0.004083	0.000943	0.005891	1.000944	2
A6	37	2.734643	0.898213	0.757326	9.824424	37	1	0	0	0	1.00	1
A7	18	1.650703	0.747359	0.571104	3.958186	18	1	0	0	0	1.00	1
Average	19 ± 9.8	2 ± 0.7	0.75±0.22	1 ± 0.2	61 ± 3.4	18.57±9.78	1.29±0.49					1.29±0.49
B1	5	1.396404	0.747856	0.867635	3.965989	5	2	0.056851	0.020163	0.082019	1.020578	2
B2	23	2.599736	0.907108	0.829131	10.76516	23	1	0	0	0	1.00	1
B3	14	1.631996	0.750932	0.618401	4.014962	14	2	0.001395	0.000283	0.002013	1.000283	2
B4	7	1.377463	0.681323	0.707876	3.137976	7	4	0.058188	0.017796	0.041973	1.018118	4
B5	12	1.717067	0.77267	0.690999	4.398897	12	1	0	0	0	1.00	1
B6	6	1.393619	0.747154	0.777794	3.954984	6	2	0.049155	0.016924	0.070916	1.017216	2
B7	12	1.810743	0.794263	0.728697	4.860575	12	1	0	0	0	1.00	1
Average	11 ± 6.21	1.7 ± 0.43	0.8 ±0.07	0.7 ± 0.09	5.01 ± 2.59	11.29±6.21	1.86±1.07					1.86±1.07
D1	15	2.164121	0.858454	0.799143	7.064865	15	2	0.001806	0.000377	0.002606	1.000377	2
D2	16	1.835379	0.786576	0.661973	4.685511	16	4	0.159168	0.063211	0.114815	1.067477	4
D3	10	2.083656	0.860267	0.90492	7.156523	10	1	0	0	0	1	1
D4	36	2.313813	0.845374	0.645682	6.467207	36	4	0.020706	0.005178	0.014936	1.005205	4
D5	16	1.798805	0.780191	0.648782	4.549399	16	5	0.12428	0.046534	0.07722	1.048805	5
D6	36	2.457644	0.864651	0.685819	7.388298	36	4	0.027907	0.007337	0.020131	1.007391	4
D7	10	1.56241	0.765524	0.678546	4.264823	10	1	0	0	0	1	1
Average	20±11.32	2.03± 0.31	0.82±0.04	0.72±0.10	5.94±1.38	19.86±11.32	3.00±1.63					3.00±1.63

	p-value						p-value					

A Vs B	0.122	0.479	0.82	0.333	0.491	0.122	0.223	0.060	0.059	0.164	0.059	0.223
A Vs D	0.824	0.753	0.421	0.549	0.876	0.824	0.021	0.084	0.104	0.292	0.107	0.021
B Vs D	0.104	0.131	0.122	0.579	0.42	0.104	0.147	0.395	0.379	0.913	0.368	0.147

A_1_- A_7_, B_1_-B_2_, D_1_-D_6_ are *Tej* sample collected from Addis Ababa (AA), Bahir Dar(BD) and Debre Markos(DM), respectively

Obs- Observed

**Table 2:** Bacterial and fungal community structure at the relative abundance < 1% (classified as others).

Bacterial Community structure at the relative abundance of < 1% (grouped as others)						
S/N	Phylum	Class	Order	Family	Genus	RA (%)
1	Proteobacteria	Gammaproteobacteria	Aeromonadales	Aeromonadaceae	Aeromonas	0.00023
2	Proteobacteria	Gammaproteobacteria	Pseudomonadales	Moraxellaceae	Enhydrobacter	7.10E-06
3	Proteobacteria	Gammaproteobacteria	Enterobacterales	Enterobacteriaceae	Enterobacteriaceae_Unclassified	0.00666
4	Firmicutes	Bacilli	Lactobacillales	Leuconostocaceae	Fructobacillus	0.00705
5	Firmicutes	Bacilli	Lactobacillales	Leuconostocaceae	Fructobacillus	7.34E-05
6	Proteobacteria	Alphaproteobacteria	Acetobacterales	Acetobacteraceae	Gluconobacter	0.00016
7	Firmicutes	Bacilli	Lactobacillales	Lactobacillales_Unclassified	Lactobacillales_Unclassified	2.13E-05
8	Firmicutes	Bacilli	Lactobacillales	Lactobacillaceae	Lactobacillus	0.00011
9	Firmicutes	Bacilli	Lactobacillales	Lactobacillaceae	Lactobacillus	0.00018
10	Firmicutes	Bacilli	Lactobacillales	Lactobacillaceae	Lactobacillus	0.00218
11	Firmicutes	Bacilli	Lactobacillales	Lactobacillaceae	Lactobacillus	0.00771
12	Firmicutes	Bacilli	Lactobacillales	Streptococcaceae	Lactococcus	0.00202
13	Firmicutes	Bacilli	Lactobacillales	Leuconostocaceae	Leuconostoc	0.00242
14	Firmicutes	Bacilli	Lactobacillales	Lactobacillaceae	Pediococcus	0.00161
15	Firmicutes	Bacilli	Staphylococcales	Staphylococcaceae	Staphylococcus	5.68E-05
16	Firmicutes	Negativicutes	Veillonellales-Selenomonadales	Veillonellales-Selenomonadales_Unclassified	Veillonellales-Selenomonadales_Unclassified	0.00012
17	Firmicutes	Bacilli	Lactobacillales	Leuconostocaceae	Weissella	0.00025

Fungal Community structure for the relative abundance of <1% (grouped as others)						

S/N	Phylum	Class	Order	Family	Genus	RA (%)

1	Ascomycota	Saccharomycetes	Saccharomycetales	Saccharomycetales_fam_Incertae_sedis	Candida	4.49E-06
2	Ascomycota	Saccharomycetes	Saccharomycetales	Phaffomycetaceae	Cyberlindnera	5.39E-05
3	Ascomycota	Saccharomycetes	Saccharomycetales	Saccharomycetaceae	Kazachstania	0.00233
4	Ascomycota	Saccharomycetes	Saccharomycetales	Saccharomycetaceae	Kazachstania	0.00048
6	Ascomycota	Saccharomycetes	Saccharomycetales	Saccharomycetaceae	Torulaspora	4.49E-05
7	Ascomycota	Saccharomycetes	Saccharomycetales	Phaffomycetaceae	Wickerhamomyces	0.00043
8	Ascomycota	Saccharomycetes	Saccharomycetales	Saccharomycetaceae	Zygosaccharomyces	0.00011

## 2 Experimental Design, Materials and Methods

### 2.1 Sample collection, transportation and storage

Twenty-one fully matured *Tej* samples were collected from Addis Ababa (lat. 8.9806, long. 38.7578), Bahir Dar (lat. 11.5742, long. 37.3614), and Debre Markos (lat. 10.3296, long. 37.7344), Ethiopia. The samples were collected from local alcohol vendors who were selected randomly based on their willingness to sell. All of the samples were collected aseptically using sterile screw cup. Besides, samples from the same locations were collected on the same day. Finally, the collected samples transported to Kyungpook National University, Korea via insulated ice box with a freezing pack. The samples that required further analysis was stored in freezer at -20 °C.

### 2.2 DNA extraction

About 40 mL of *Tej* samples were centrifuged at 3200 rpm for 20 m to harvest the highest cell concentration. The microbial DNA was then extracted from the sediment via QIAamp PowerSoil Pro Kit (QIAGEN, Germany) by following manufacturer protocol. The final concentration of the extracted microbial DNA was checked by Qubit 2.0 Fluorometer (Life Technologies, USA).

### 2.3 16SrRNA sequencing

Amplicon sequencing for each sample was performed using a barcode set of Nextera Library Preparation Kit (Illumina Inc., USA). The hypervariable (V4 -V5) region of 16S rRNA gene was PCR amplified by using 515F (GTGNCAGCMGCCGCGGTAA) as the forward-inner primer and 907R (CCGYCAATTYMTTTRAGTTT) as the reverse-inner primer [2]. The PCR amplifications by thermocycler (Mastercycler Nexus GSX1, Eppendorf, Germany) were performed in two phases. The first PCR was run at the condition of 95 ℃ for 5 min of pre-denaturation, followed by 15 cycles of 95 ℃ for 30 s of denaturation, 60 ℃ for 30 s of annealing, 72 ℃ for 30 s of extension, and 72 ℃ for 5 min of final extension [3]. The reaction mixtures were composed of 1 µL (1 µM) of reverse inner primer, 1 µL (1 µM) of forward inner primer, 2 µL DNA template, 25 µL Emerald Amp PCR Master Mix (Takara Co., Ltd., Japan). The total volume of the PCR reaction mixture was then adjusted to become 50 µL by sterilized distilled water (SDW). The second PCR was conducted under the same running conditions as the first, by adding bar code primers and 2 µL of first PCR amplified DNA templets. These PCR amplified products were then multiplexed to 100 ng/µL into the single product via measuring the DNA concentration. Finally, amplified and barcoded DNA having 550 bp of size were selected using AMPure XP for PCR Purification (BECKMAN COULTER Inc., USA) for further downstream procedures.

### 2.4 Internal transcribed spacer (ITS) sequencing

Fungal internal transcribed (ITS2) regions were targeted for amplification using the primers of ITS86F (GTGAATCATCGAATCTTTGAA) and ITS4 (TCCTCCGCTTATTGATATGC) [4,5]. The first PCR amplification was performed at a condition of 95 °C for 5 min, followed by 30 cycles of 95 °C for 30 s, 58 °C for 30 s, 72 °C for 30 s, and finally 72 for 5 min (Jung et al., 2020). The second amplification was also carried out in the same condition as it was done for the first one. The reaction mixtures for the above mentioned two PCR amplifications were composed of 1 µL (1 µM) of reverse primer, 1 µL (1 µM) of forward primer, 2 µL DNA template, 25 µL Emerald Amp PCR Master Mix, 21 µL sterilized distilled water (SDW).

### 2.5 High-throughput sequencing

Before high-throughput sequencing, the amplicon library size, and quality and quantity were double-checked via Agilent 2100 Bioanalyzer (Agilent Technologies Inc., USA). Then amplicon libraries were directly subjected to the Illumina MiSeq platform by following the manufacturer's instructions. The base calling and image analysis were performed using MiSeq Control Software (MCS) which is installed in the Illumina MiSeq instrument.

### 2.6 Bioinformatics and statistical analysis

Quantitative insights into microbial ecology 2 (QIIME2) was used for the analysis of raw sequence FASTQ data. Filtering, trimming, and denoising of the raw sequences were performed via DADA2 to obtain amplicon sequence variants (ASV) [6]. Taxonomic identification of bacterial and fungal communities, the SILVA and UNITE reference databases were utilized, respectively. Vegan package was used for alpha diversity analysis of Shannon, Chao1, Simpson, Evenness, and InvSimpson. Meanwhile, the linear discriminant analysis effect size (LEfSe) and principal coordinates of analysis (PCoA) plots were performed via Web-based Calypso and RStudio 4.0.3. All of these microbiome data analyses were performed by applying a non-parametric Kruskal–Wallis tests with alpha value of less than 0.05 to detect significant difference in microbiome features between the group of collected sample.

## CRediT authorship contribution statement

**Eskindir Getachew Fentie:** Conceptualization, Methodology, Formal analysis, Investigation, Data curation, Writing – original draft, Visualization. **Minsoo Jeong:** Investigation, Software, Visualization. **Shimelis Admassu Emire:** Conceptualization, Writing – review & editing, Supervision. **Hundessa Dessalegn Demsash:** Conceptualization, Writing – review & editing, Supervision. **Min A Kim:** Investigation. **Hwang-Ju Jeon:** Investigation. **Sung-Eun Lee:** Supervision. **Setu Bazie Tagele:** Methodology. **Yeong-Jun Park:** Methodology. **Jae-Ho Shin:** Conceptualization, Writing – review & editing, Resources, Supervision.

## Declaration of Competing Interest

The authors declare that they have no known competing financial interests or personal relationships that could have appeared to influence the work reported in this paper.

## Data Availability

Alpha diversity and Microbial community tables (Original data) (Science Data Bank).
